# Physiological Responses to Virtual Reality‐Based Stress Regulation and Relaxation Interventions: A Systematic Review

**DOI:** 10.1002/smi.70164

**Published:** 2026-03-14

**Authors:** Lisanne M. Robbemond, Matthijs L. Noordzij, Catheleine M. G. van Driel, Wim Veling

**Affiliations:** ^1^ Department of Psychiatry University of Groningen University Medical Center Groningen Groningen the Netherlands; ^2^ Department of Psychology University of Twente Health and Technology Enschede the Netherlands; ^3^ VRelax BV Groningen the Netherlands

**Keywords:** heart rate variability, psychophysiology, relaxation, stress, systematic review, virtual reality

## Abstract

Physiological markers are increasingly used to monitor individuals undergoing Virtual Reality (VR) stress interventions. However, several challenges arise when employing these markers to assess the impact of VR stress interventions. This systematic review synthesises and critically discusses the literature on VR stress interventions across contexts, study designs using physiological markers, marker usage, measurement setups, trends in interpreting data for stress recovery, and meanings assigned to marker changes. This review follows the PRISMA guidelines. EMBASE, IEEE Xplore, PsycINFO, PubMed, and Web of Science were searched until 12th March 2024. Studies were included if they used a head‐mounted display (HMD) VR stress intervention and evaluated at least one physiological marker. The quality of studies was evaluated using the Evaluation Public Health Practice Project tool. Data were systematically extracted and summarised thematically and narratively. This review was registered with PROSPERO ID: CRD42024510162. Out of 11,479 articles identified, 69 studies were included: 59 conducted in the general population, four in mental healthcare, and six in the professional context. Study characteristics, experiment characteristics, and physiological outcomes were extracted. Most studies featured nature‐based experiences, referenced Attention Restoration Theory (ART) and Stress Reduction Theory (SRT), and induced stress with mental arithmetic tasks or the Trier Social Stress Test (TSST). Many studies lacked theoretical grounding, had small sample sizes, conducted only a single VR session in a laboratory setting, and had low quality. Physiological markers and timepoints varied widely, with heart rate (HR), HRV (RMSSD, SDNN, LF, HF), and blood pressure being the most common. Fifty‐six studies found that VR interventions reduced stress as measured by physiological markers. However, methodological inconsistencies limited their findings. Future research should prioritise ambulant measurements to improve real‐world applicability and assess long‐term effects. Additionally, standardising marker selection and measurement protocols is necessary to enhance validity and comparability, thereby strengthening evidence of VR stress interventions.

**Trial registration:**

PROSPERO ID: CRD42024510162

## 1 Introduction

Stress is a multidimensional phenomenon affecting human health and well‐being. Virtual Reality (VR) is a promising tool for stress reduction and management through immersive, controlled environments. Numerous VR studies have included assessments of physiological stress markers; however, interpretations of these markers often remain ambiguous. The present systematic review focuses on non‐invasive physiological markers as key outcomes of VR stress interventions, strongly emphasising measuring stress recovery. These include cardiac measures (e.g., heart rate (HR), heart rate variability (HRV)), electrodermal activity, respiratory rate, blood pressure, and salivary cortisol, all of which can be collected continuously or at a discrete time point without requiring invasive procedures such as surgical implantation or venipuncture. Reliable markers could advance VR stress interventions through passive monitoring and biofeedback. Despite growing interest, this field is hindered by several unresolved issues. Unresolved issues include a lack of consensus on the definition of stress in experimental contexts, accounting for individual variability in stress responses, interpreting physiological changes accurately, and establishing meaningful norm scores for effective stress recovery and management.

### 1.1 Virtual Reality for Relaxation or Stress Induction

Numerous VR systems have been developed to manage stress in various settings, including the military, healthcare, and mental healthcare. Among these, virtual natural environments have shown particular effectiveness in promoting relaxation and reducing perceived stress across diverse populations (Nijland et al. 2021; Riches et al. 2021, 2023; Veling et al. 2021). The stress‐reducing effect is supported by environmental psychology theories: the Stress Recovery Theory (SRT) proposes that natural environments reduce stress by eliciting positive emotions and lowering physiological arousal (Ulrich 1983; Ulrich et al. 1991). Similarly, the Attention Restoration Theory (ART) posits that natural settings restore depleted attention (R. Kaplan and Kaplan 1989; S. Kaplan 1995).

In experimental research, the VR‐Trier Social Stress Test (VR‐TSST) is commonly used to simulate social stress through public speaking and arithmetic tasks (Helminen et al. 2019, 2021; Kirschbaum et al. 1993; Shiban et al. 2016). In clinical contexts, VR stress inoculation training (VR‐SIT) and VR stress management training (VR‐SMT) are prominent. VR‐SIT can be utilised for a wider range of stress‐related settings by incorporating exposure to various stressors and allowing individuals to build resilience and coping skills in response to stress (Meyerbröker and Emmelkamp 2010; Parsons 2015). VR‐SMT focuses on reducing stress and promoting relaxation through immersive experiences using 360‐degree videos of calming natural scenes combined with guided relaxation, mindfulness, and visualisation techniques (Botella et al. 2017; Pallavicini et al. 2016; Riva et al. 2019; Veling et al. 2021). It is important to distinguish VR stress interventions from VR exposure therapy (VRET), which targets anxiety disorders and specific phobias through exposure and fear extinction paradigms. VRET has strong empirical support (Botella et al. 2017; Freitas et al. 2021; Krijn et al. 2004). However, VRET and VR stress interventions differ fundamentally in their clinical target and therapeutic mechanisms. VRET intentionally induces fear and anxiety through controlled exposure to feared stimuli, enabling new learning (e.g., expectancy violation) through habituation and progressive loss of fear (Foa 2011). While some degree of anxiety and physiological arousal commonly accompanies effective exposure, contemporary inhibitory learning accounts suggest that the quality of learning (e.g., violated threat expectancies) is more critical than achieving a specific level of autonomic activation or within‐session habituation (Craske et al. 2008, 2014). In contrast, VR stress interventions aim to reduce stress responses and promote relaxation, with decreased physiological arousal as the desired therapeutic outcome. These approaches target different clinical conditions (anxiety and fear vs. stress) and have opposite expectations for physiological responses. The present review focuses specifically on VR interventions designed to address stress and promote relaxation, rather than exposure‐based therapies for anxiety disorders. This distinction is critical for the appropriate interpretation of physiological data, as the same physiological pattern may indicate therapeutic progress in one context but therapeutic failure in another.

### 1.2 Definition of Stress and the Physiological Stress Response

Understanding stress is complex due to individual differences in sensitivity, reactivity, coping mechanisms, and perceptions, as well as the lack of consensus on a unified definition (Epel et al. 2018; H. G. Kim et al. 2018; McEwen and Gianaros 2010; Pallavicini and Mantovani 2017; Schneiderman et al. 2005; Selye 1956). Stress responses involve physiological, psychological, and behavioural responses that challenge coping resources to events, ranging from significant life events to minor daily hassles, and include both acute and chronic forms.

Short‐term stressors activate the hypothalamic‐pituitary‐adrenal (HPA) axis and autonomic nervous system (ANS), comprising the sympathetic (SNS) and parasympathetic (PNS) branches. The SNS is associated with “fight‐or‐flight” responses – elevated heart rate (HR), blood pressure, and energy mobilisation ‐ while the PNS supports “rest‐and‐digest” functions and recovery. However, these systems can co‐activate or co‐inhibit, challenging the traditional reciprocal model, a concept reflected in models of cardiac autonomic regulation (Berntson et al. 2008). A nuanced interaction between the HPA axis and the ANS is central to interpreting physiological stress responses, primarily through heart rate variability (HRV) and related ANS markers.

Chronic stressors activate similar systems but often lead to dysregulation, including disrupted cortisol rhythms. This dysregulation is associated with increased risks for chronic pain, cardiovascular disease, mental health issues, and reduced workplace functioning (Abdallah and Geha 2017; Cohen et al. 2007; Epel et al. 2004, 2018; Kivimäki and Steptoe 2017; Lupien et al. 2009; Marin et al. 2011; Monroe and Harkness 2005; Phillips et al. 2007). Over time, cumulative stress effects, referred to as allostatic load, can impair homoeostasis and overall health by overburdening stress response systems.

### 1.3 Measuring Stress Responses With Physiological Markers

Physiological markers offer objective insights into stress responses and recovery, supporting the evaluation of VR stress interventions. Implicit assessments, such as HRV, skin conductance, and cortisol, capture physiological stress responses passively (Marín‐Morales et al. 2020; Moon and Lee 2017), while explicit methods, like questionnaires and interviews, assess subjective experiences (Brosschot et al. 2006; Cohen et al. 1983).

Cardiovascular activity, a key focus, is measured using electrical (ECG), optical (PPG), or blood pressure (BP) methods. HRV reflects the dynamic interplay of SNS and PNS branches of the ANS and serves as a marker of autonomic regulation and stress reactivity, rather than a direct marker of the stress response (Quigley et al. 2024). In general, lower HRV may indicate autonomic inflexibility and potential health risks (Thayer et al. 2010). However, the relationship between the stress response and HRV parameters remains nuanced, as HRV captures multiple components of autonomic balance rather than a single continuum from “relaxed” to “stressed”. This complexity arises because different HRV indexes represent both sympathetic activation and parasympathetic withdrawal, and various HRV parameters correspond to distinct facets of these dynamics (H. G. Kim et al. 2018; Malik 1996).

To capture these dynamics, HRV can be quantified in the time domain and frequency domain. Time‐domain indices, such as the standard deviation of normal‐to‐normal intervals (SDNN) and the root mean square of successive differences (RMSSD), provide overall and short‐term variability, respectively. In the frequency domain, high‐frequency (HF) power reflects respiratory sinus arrhythmia and vagal modulation, making it a relatively specific index of PNS activity. Both SNS and PNS influence low‐frequency (LF) power, therefore representing mixed autonomic control. The LF/HF ratio has been interpreted as an index of sympathovagal balance; however, this is strongly debated due to the mixed origins of LF power and the ratio's limited physiological specificity (Billman 2013; Quigley et al. 2024). Thus, HRV offers a multidimensional perspective on cardiovascular regulation under stressors and recovery, and is central to the outcomes reported in this review.

Electrodermal activity (EDA), or galvanic skin response (GSR), measures SNS activity, making it valuable for understanding stress and arousal in this review. It includes skin conductance level (SCL), which indicates tonic, baseline skin conductance over time, and skin conductance response (SCR), which reflects rapid, phasic reactions to stimuli. These metrics complement HRV by capturing SNS‐driven arousal.

Respiratory activity, another physiological marker, is assessed by measuring the respiratory rate (i.e., the number of breaths taken per minute), providing additional context for stress‐related autonomic regulation. Complementary measures such as blood or saliva tests for cortisol and dehydroepiandrosterone (DHEA) offer a broader perspective on the neuroendocrine stress response, indicating autonomic and neuroendocrine dynamics in response to stress and recovery. Notably, physiological stress markers exhibit different normative values and reactivity patterns in children compared to adults, which may complicate cross‐age comparisons (Evans et al. 2021; Mikneviciute et al. 2023).

Despite growing research on VR systems for relaxation management, gaps remain in how physiological markers are utilised and interpreted. While head‐mounted VR shows promise in general and mental health populations, few studies focus specifically on physiological markers (Riches et al. 2021, 2023). Challenges include inconsistent selection, measurement, and reporting of markers, which limit standardisation. Individual variability and contextual influences further complicate interpretation. Although guidelines like the Guidelines for Reporting Articles on Psychiatry and Heart Rate Variability (GRAPH) (Quintana et al. 2016) support HRV reporting in psychiatry, no equivalent exists for VR‐based interventions, which involve unique contexts and applications. A more structured approach is needed to enhance the reliability and comparability of findings in this emerging field.

### 1.4 Aim of the Current Systematic Review

This review provides a comprehensive overview of physiological markers in VR‐based stress interventions. VR stress interventions are defined as any VR experiences aimed at reducing psychological or physiological stress, including those labelled as “relaxation” when stress reduction is a stated goal. While physiological markers provide objective indices of autonomic and neuroendocrine activity, they represent only one dimension of the stress response and cannot be equated with subjective distress or clinical outcomes (Taschereau‐Dumouchel et al. 2020). Physiological arousal patterns do not map directly onto conscious emotional experiences. Our emphasis on physiological monitoring reflects both their prominence in current VR stress research and the need for critical examination of how these measures are interpreted and used. This review explores how these markers are evaluated to assess the effectiveness of VR interventions for stress management. Primary outcomes are cardiovascular markers: HR, HRV, including RMSSD, SDNN, LF, HF, LF/HF ratio, and blood pressure (BP). Secondary markers are markers from other physiological systems: EDA, including SCL and SCR, respiratory rate (RR), and cortisol.

This review addresses the following questions.

1. What types of VR stress interventions are delivered in various contexts?
2. What theoretical frameworks are used for selecting physiological markers?
  - What hypotheses are stated regarding improvements in physiological stress caused by VR interventions?
3. Which physiological markers and outcomes are used to evaluate VR stress interventions?
  - At which time points and for what duration are physiological markers measured?
  - In which setting are these measurements conducted?
4. What methods and decision rules are used to interpret these markers?
  - What meaning do researchers assign to changes in physiological markers in VR research?
  - What are the trends in the interpretations of physiological data across various contexts when evaluating the effectiveness of VR interventions?
5. What is the recommended research setup and design for utilising physiological markers to evaluate the effectiveness of VR interventions?

## 2 Methods

The systematic review methodology followed the Preferred Reporting Items for Systematic Reviews and Meta‐Analysis (PRISMA 2020) guidelines (Page et al. 2021). A protocol was developed in accordance with the PRISMA‐P (Preferred Reporting Items for Systematic Review and Meta‐Analysis Protocols) guidelines (Moher et al. 2015). This review was registered in PROSPERO International Prospective Register of Systematic Reviews on 19.02.2024 (ID CRD42024510162; https://www.crd.york.ac.uk/prospero/display_record.php?ID=CRD42024510162).

### 2.1 Search Strategy

On 12 March 2024, five databases (EMBASE, IEEE Xplore, PsycINFO, PubMed, and Web of Science) were searched for relevant literature. The general keyword search terms were: (VR OR virtual reality OR virtual reality exposure therapy) AND (psychological stress OR physiological stress OR respiratory rate OR blood pressure OR hydrocortisone OR heart rate OR galvanic skin response OR dehydroepiandrosterone). VRET was included to ensure comprehensive coverage, though VRET studies exclusively focused on exposure therapy without an explicit stress‐reducing component were excluded, as this review specifically focuses on stress‐reducing interventions. A detailed list of search strings is available as a supplement (Appendix A). One study was identified through a manual search.

No restrictions were placed on study design during the search. Articles published before March 11, 2024, and in English were considered. Inclusion criteria were: (1) evaluated a VR stress intervention targeting stress reduction or relaxation in at least one experimental group; (2) evaluated at least one physiological marker of stress response; (3) general or mental health population; (4) adults; (5) head‐mounted display. Exclusion criteria were: (1) VR system not aimed at stress management or relaxation (e.g., studies using VRET focused solely on exposure therapy); (2) augmented reality, mixed reality, or no head‐mounted display; (3) VR system for medical complaints or skills training; (4) invasive physiological measures; (5) full text unavailable; (6) individuals under 18 years. Furthermore, research solely focused on qualitative analysis and results, and review articles were excluded.

### 2.2 Procedure

The results of the literature search were uploaded to Mendeley (version 1.19.8) to remove duplicates. To make the selection process faster, transparent, and easily replicable, records were uploaded to ASReview, a machine‐learning‐assisted screening tool (ASReview LAB developers, 2025; Van De Schoot et al. 2021). The software presents the most relevant record based on prior selection, refining the model with each decision. A simulation study showed that reviewing just 8%–33% of the total records can identify up to 95% of relevant studies (Van De Schoot et al. 2021).

Titles and abstracts were screened in ASReview for eligibility using predefined criteria and the SAFE procedure's machine learning and human‐in‐the‐loop techniques (Boetje and Van De Schoot 2024). Screening began with a random training set, where at least 1% (65 records) was reviewed, ensuring the identification of at least one record. Two reviewers (authors LMR and MLN) repeated this process four times. The fraction of relevant records in the training set (FFR_t) guided the transition to the next phase.

Phase two aimed to maximise the efficient identification of relevant records. The ASReview algorithm, employing a Naïve Bayes classifier, TD‐IDF feature extraction, and certainty‐based sampling, continued screening until all stopping heuristics were met: identification of key papers, screening of at least twice RR_t (FFR_t * total records), review at least 10% of the dataset, and absence of relevant records in the final 50 screened.

Phase three utilised a different machine‐learning model (fully connected neural network as classifier and Sentence BERT as feature extraction and dynamic resampling) to find more relevant records. Prior knowledge from one reviewer (author LMR) informed training, with screening ceasing upon failure to identify new relevant records in the last 50 screened.

Finally, phase four involved expert validation (author CMGvD), which reviewed an unlabeled random subset, including the 10 highest‐ and lowest‐ranked records per reviewer, concluding when no further relevant records emerged.

Full‐text screening and data extraction were done using Covidence. Consensus between the two reviewers (authors LMR and MLN) was a prerequisite for inclusion, and differences in judgement were resolved by discussion. From each included study, the study characteristics (authors, country, sample size, participant group, context, research design), experiment characteristics (scenario, frequency, duration per condition), and physiological outcomes (parameters, timepoints, setting) were extracted.

Meta‐analyses were conducted when five or more studies examined the same physiological marker using a consistent study design. This threshold was chosen because estimating between‐study variance with fewer than five studies can be unstable and potentially misleading (Jackson and Turner 2017). In cases where these criteria were not met, harvest plots were used to visually synthesise the direction of effects across studies in a structured manner.

### 2.3 Quality Assessment

The Evaluation of Public Health Practice Project (EPHPP) made the Quality Assessment Tool for Quantitative Studies (QATA), which was used to evaluate the quality of the included studies (Thomas et al. 2004). This tool assesses six key domains: selection bias, study design, confounders, blinding, data collection methods, and withdrawal and drop‐outs. Each domain was assigned a score of “strong” (1), “moderate” (2), or “weak” (3). A global quality rating was assigned based on the domain scores: strong (no weak ratings), moderate (one weak rating), or weak (two or more weak ratings). The EPHPP tool was chosen for its applicability to diverse study designs and its structured, transparent approach to quality appraisal.

## 3 Results

### 3.1 Study Selection and Characteristics

The final search on March 12, 2024, identified 11,479 records, with 6489 unique entries. After title and abstract screening, 140 full texts were assessed, and 69 studies met the inclusion criteria. Most of the studies were conducted in the United States, Germany, Australia, Italy, and South Korea, with a total of 3186 participants. Fifty‐six studies focused on the general population, including healthy adults, university students, and individuals reporting high‐stress levels. Four studies were conducted in mental healthcare settings, defined as studies targeting patients with psychiatric conditions such as generalised anxiety disorder (GAD). Seven studies were conducted in the professional context, involving the working populations (including clinicians, factory workers, and veterans). The diversity in population type reflects the differences in baseline stress levels and intervention applicability, which may moderate the observed effects of VR interventions.

**FIGURE 1:**
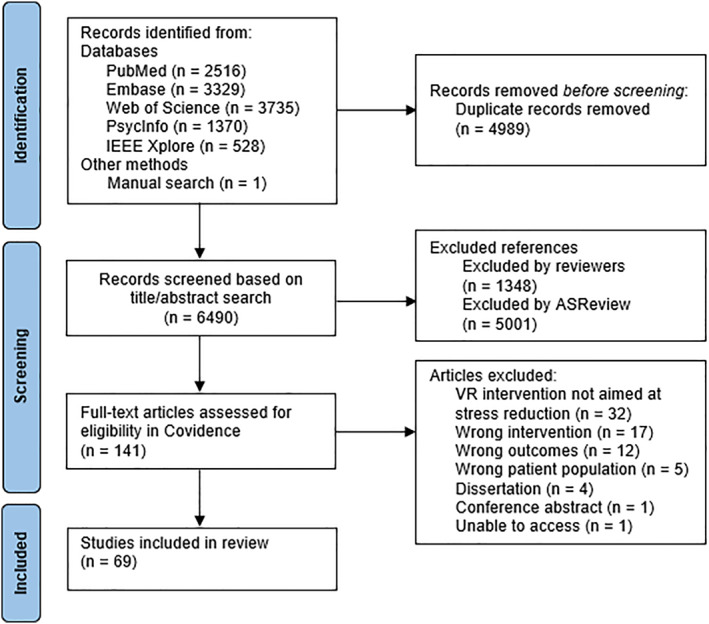
PRISMA flowchart for the study selection process.

Quality assessment rated 62 studies as ‘weak’ in over two domains, leading to a weak global quality rating. This was mainly due to selection bias (e.g., convenience sampling), study design (i.e., small sample size, unclear randomisation), and confounders (e.g., no indication of control for confounding variables). Study selection details are shown in Figure 1. Table 1 summarises the included studies, distinguishing between single‐use interventions (*n* = 50) and multi‐session VR interventions or study designs involving repeated exposure to the same or varied VR systems (*n* = 19). If the duration of physiological marker measurements was specified, it is indicated within parentheses. The studies employed between‐subjects (*n* = 21), within‐subjects (*n* = 34), or mixed design (*n* = 14), comparing within‐ and between‐subjects differences.

**TABLE 1:** Characteristics of included studies.

Study characteristics			Experiment characteristics	Physiological outcomes		
Study	*N* Sample	Type of study	Scenario; frequency; duration per condition	Setting; measures	Summary of findings	EPHPP
(Adhyaru and Kemp 2022); UK	49; clinicians	Experiment; within	1. Virtual meadow with interaction; 1; 600s	Ambulatory; HR	**Baseline (t1), pre‐intervention (t2), post‐intervention (t3):** t2→t3, time effect HR, decreased HR.	3
(Aganov et al. 2020); Ukraine	94: Healthy with moderate stress	Crossover RCT; within	1. Virtual beach walk with binaural effect and walking pace of the cat synchronized with resting HR; 1; 300s 2. Virtual beach walk with cat without binaural effect synchronization; 1; 300s	Laboratory; HR, RMSSD, SDNN, HF, LF/HF, SBP, DBP, TP, RR	**Baseline (300s, t1), stress task (300s, t2), intervention (300s, t3), post‐intervention (300s, t4):** t1, no differences in SBP, DBP, RR, and HR. t2→t4, decreased HR and LF/HF, and increased RMSSD in both conditions. Larger decreased LF/HF and increased RMSSD in condition 1 than 2. t4, difference in HF between conditions, increased HF in condition 1 not 2. No difference in SDNN, SBP, DBP, TP, and RR. Condition and time sequence did not influence the HR, RMSSD, SDNN, LF/HF, SBP, DBP, TP, and RR.	2
(Ahmaniemi et al. 2017); Finland	4; Employees	Crossover experiment; within	1. Virtual videos of a tropical beach, the Alps, and Portugal; 3; 1200s 2. Audio‐only relaxation exercises; 3; 1200s	Laboratory; HR, LF, HF, LF/HF, SBP, DBP, SCL	**Baseline (30 × 60s, t1), session (1200s, t2), post‐session (30 × 60s, t3):** t2, decreased HR and SCL. Lower HR and SCL in condition 2 than 1. LF and HF are influenced by subject and session, LF/HF only by subject. **Baseline (t1), pre‐session (t2), post‐session (t3):** DBP and SBP influenced by subject.	3
(Alyan et al. 2021); Malaysia	20; healthy students	Randomized experiment; mixed	1. Virtual realistic forest with interaction; 1; 300s 2. Virtual dreamlike environment with interaction; 1; 300s	Laboratory; HR, SCL	**Baseline (t1), post‐stress task (t2), pre‐intervention (t3), post‐intervention (t4):** t1→t2, increased HR and SCL in both conditions. t3→t4, decreased HR and SCL in both conditions.	3
(Anderson et al. 2017); USA	18; healthy	Counter‐balanced RCE; within	1. Virtual environment of various locations in Ireland; 1; 900s 2. Virtual environment of various locations in Australia; 1; 900s 3. Empty indoor classroom; 1; 900s	Laboratory; LF, HF, LF/HF, EDA	**Baseline (120s, t1), stress task (120s, t2), begin intervention (120s, t3), mid‐intervention (120s, t4), end intervention (120s, t5):** Time effect on LF for conditions 1 and 3, lower at t2 than t3. Time effect on HF for conditions 2 and 3, t2 differed from t3‐t5. Time effect on LF/HF for conditions 2 and 3, condition 2, difference between t3, t4, and t5. Condition 3, highest at t2 than t3‐t5. Time effect on EDA, overall decreased. Condition 2 dropped below t1.	3
(Blum et al. 2019); Germany	60; healthy employees	RCE; mixed	1. VR‐based HRV‐BF, virtual beach, continuous BF connected to cloud coverage, dichotomous BF connected to lamps; 1; 600s 2. Standard HRV‐BF; visual abstract, continuous BF and background music; 1; 600s	Laboratory; HR, RMSSD	**Questionnaire 1 (t1), stress task 1 (t2), questionnaire 2 (t3), intervention (600s, t4), questionnaire 3 (t5), stress task 2 (t6), questionnaire 4 (t7):** No effect of condition or time x condition on HR. Time effect on HR. t2, increased HR, decreased HR stepwise t3→t7. Time effect on RMSSD, increased RMSSD at t4 compared to others. No condition x time effect on RMSSD.	3
(Blum et al. 2020); Germany	72; healthy students	RCE; between	1. VR RBF, abstract nature, colour changes based on respiration; 1; 420s 2. Virtual breathing exercise, abstract nature, colour changes at random; 1; 420s	Laboratory; RMSSD, LF, HF, app. RR	**Intervention (420s):** Higher RMSSD, LF, and lower app. RR, in condition 1 than 2. No difference in HF.	3
(Calogiuri et al. 2018); Norway	34; healthy	Counter‐balanced randomized experiment; within	1. Outdoor walk in nature; 1; 600s 2. Sedentary exposure to IVE video; 1; 600s 3. Treadmill walks with exposure to the same IVE video; 1; 600s	Laboratory; HR M, HR max	**Intervention (600s):** HR mean and max, differences across conditions. Higher HR mean and HR max in conditions 1 than 2, not 3. Condition 3 had a higher HR mean and max compared to 2.	3
(Cawley and Tejeiro 2024); UK	67; students	Randomized experiment; mixed	1. Virtual mindfulness exercises in nature environment; 1; 600s 2. Audio of mindfulness and breathing exercise; 1; 600s 3. Coloring; 1; 600s	Laboratory; HR	**Pre‐intervention (t1), intervention (300s, t2), post‐intervention (t3):** t1, no differences in HR. t1→t3, increased HR in condition 1, not 2 and 3. Differences between conditions 1 and 3, 2 and 3, not 1 and 2.	2
(Chan et al. 2023); Singapore	Study 1:12; students study 2:26; elderly	Counter‐balanced experiment; within	1. Virtual nature walk; 1; 300s/180s 2. Virtual urban walk; 1; 300s/180s	Laboratory; IBI, RMSSD	**Study 1: Baseline (60s, t1), intervention (300s, t2):** Time x condition effect on IBI, no time or condition effects. t1→t2, increased IBI for condition 1, not 2. No time, condition, or time x condition effects on RMSSD. **Study 2: Baseline (60s, t1), intervention (180s, t2):** Irregularities in data, the remaining sample is too small.	3
(Chavez et al. 2020); USA	30; Homeless youth	Pilot RCT; between	1. Virtual guided meditation in natural scene; 1; 600s 2. Audio meditation; 1; 600s 3. VR imagery, viewing historic images and text; 1; 600–900s	Laboratory; cortisol	**Baseline (t1), baseline + 5 min (t2), post‐intervention (t3), post‐intervention + 15 min (t4):** No changes from t2→t3 and t2→t4 in all conditions, no differences between conditions.	2
(Chen and Li 2022); Taiwan	15; healthy	Randomized experiment; between	1. Virtual environments of mountaintop and sea, in random order; 1; 240s 2. 2D videos of nature; 1; 240s 3. Meditation, instructions to imagine mountains; 1; 240s.	Laboratory; HR	**Baseline (120s, t1), stress task (120s, t2), respiration (120s, t3), intervention (240s, t4):** t1→t2, increased HR in all conditions. t2→t4, decreased HR in all conditions, most in condition 1.	3
(Crosswell and Yun 2022); USA	5; students	Longitudinal experiment; within	1. Virtual guided meditation, audio guided meditation; or unguided self‐meditation, in random order; x; x	Ambulatory and laboratory; HR	**Baseline (2 weeks, t1), pre‐session (300s, t2), post‐session (300s, t3):** t2→t3, decreased HR across sessions, effect varied by participant. No differences between meditation types. No differences in HR over 6 weeks.	3
(De Jesus Junior et al. 2023); Canada	24; PTSD	Longitudinal experiment; within	1. Virtual beach and cardiac coherence exercise, virtual rocky seaside and audio‐guided meditation, or only mountain lake. In random order; 12; 900s	Laboratory; HR, IBI, RMSSD, SDNN	**^a^** **Pre‐intervention 1 (pre/post 60s, t1), pre‐intervention 2 (pre/post 60s, t2), post‐intervention (pre/post 60s, t3), 3‐month follow‐up (pre/post 60s, t4):** Pre→post at each t; lower HR, higher IBI, RMSSD, and SDNN.	3
(El‐Qirem et al. 2023); Jordan	90; students	Experiment; within	1. VR therapy, river ride; 8; 900s	Laboratory; pulse	**Pre‐session (t1) ‐ post‐session (t2):** t1→t2, decrease pulse	3
(Frigione et al. 2022); Italy	34; healthy	Counter‐balanced experiment; within	1. Virtual forest; 1; 120s 2. Virtual living room; 1; 120s	Laboratory; HR, SCL, RR	**Baseline (120s, t1), intervention (120s, t2), cognitive task (t3):** No effect on HR and RR. t2, higher SCL in condition 1 than 2. Higher SCL in first exposure than in second. No order x condition effect. t3, higher SCL in condition 2. No order or order x condition effects.	3
(Gerber et al. 2019); Switzerland	45; healthy	Crossover randomized observational experiment; within	1. Virtual island with beach; 1; 600s 2. Virtual downtown from busy to quieter; 1; 600s 3. Nature documentary; 1; 600s	Laboratory; HR, RR, mean BP	**Intervention (600s)**: Decreased HR in condition 1. Negative correlation time and HR in all conditions. Decreased RR in conditions 1 and 2, increased RR in condition 3. Negative correlation time and RR for condition 1. No effect on mean BP.	3
(Gonzalez et al. 2021); Australia	50; students	Experiment; between	1. Augmented‐virtual (AV), virtual human gives relaxation tips, match with real‐world objects; 1; x 2. Virtual (V), virtual human gives relaxation tips, no match with real‐world objects; 1; x	Laboratory; HR	**Baseline (t1), post‐stress task (t2), post‐intervention (t3):** Time effect on HR. t2→t3, different HR when combining conditions, decreased HR in condition 1.	3
(Gu et al. 2021); China	30; students	RCE; between	1. Virtual beach and sea; 1; 900s 2. 2D environment of beach and sea; 1; 900s	Laboratory; EDA	**Stress task (100s, t1), begin intervention (300s, t2), mid‐intervention (300s, t3), end intervention (300s, t4):** Time and time x condition effect on EDA. t1, increased EDA, t2→t4 continued to decrease below t1 for both conditions. t4, slight EDA increase for condition 2.	3
(Ho et al. 2023); Taiwan	40; factory workers	Non‐randomized experiment; between	1.Virtual nature videos including parks, hiking trails, forest paths, and bikeways; 12; 1800s 2. No intervention, free to do any activities of their choosing; 12; 1800s	Ambulatory; HR, SBP, DBP, SDNN, LF, HF, LF/HF, TP	**^b^** **Baseline (300s, t1), pre‐session (300s, t2), post‐session (300s, t3):** t1, larger SBP and DBP in condition 1 than 2. t2→t3, Condition x time effect on SBP and DBP, increased SBP and DBP for condition 2, time effect on DBP, increased in both conditions. Condition effect on SDNN, time effect on TP, time and condition x time effect on LF and HF.	3
(Ilioudi et al. 2023); Sweden	60; psychiatric patients	Experiment; mixed	1. Virtual relaxation room with nature environments relaxation exercises; x; x 2. Physical sensory room with relaxing stimuli; x; x	Ambulatory; HR, SBP, DBP	**Pre‐session (t1), post‐session (t2):** t1→t2, only time effect HR, decreased HR. Only condition effect on SBP, decreased SBP in condition 2. No time, condition, and time x condition effects DBP.	3
(Ishaque et al. 2020); Canada	14; healthy	Experiment; within	1. Virtual fishing game with biofeedback; 1; x	Laboratory; HR, RMSSD, SDNN, pNN50, SD1, SD2, ApEN, VLF, LF, HF, LF/HF, GSR M, GSR SD, RR	**^c^** **Baseline (t1), stress task (t2), stress task 2 (t3), intervention (t4):** t1→t3, **i**ncreased SD1, SD2, ApEN, LF, and decreased HF. t4, decreased HR, ApEN, LF, LF/HF, GSR M, and increased HF.	3
(Jeong et al. 2022); South Korea	31; healthy	Randomized experiment; mixed	1. Virtual DB and PMR and practice; x; x 2. Worksheet with instructions on DB and PMR and practice; x; x	Laboratory; RMSSD, SDNN, nLF, nHF, LF/HF	**Pre‐session (t1), post‐session (t2):** t1→t2, no changes in SDNN, RMSSD, nLF, nHF, LF/HF or differences in their change rate for both conditions.	3
(Jin et al. 2023); Taiwan	30; healthy	RCE; between	1. Virtual forest with handle interaction; 1; 300s 2. Virtual forest with gesture interaction; 1; 300s 3. Virtual forest without interaction; 1; 300s	Laboratory; HR, LF/HF	**Baseline (120s, t1), stress task (60s, t2), intervention (300s, t3):** t1→t2, increased HR in all conditions. t2→t3, decreased HR in all conditions, difference between conditions 1 and 2. No difference in LF/HF between conditions.	3
(S.‐H. Jo et al. 2021); South Korea	60; students	RCE; between	1. Virtual environment of forest walk; 4; 300s 2. 2D video of forest walk; 4; 300s 3. No treatment; x; x	Laboratory; RMSSD, SDNN, LF, HF	**^b^** **Pre‐intervention period (150s, t1), post 1‐week intervention period (150s, t2):** t2, differences between conditions 1 and 2, higher HF in condition 1. t2, differences between conditions 1 and 3, higher HF, SDNN, and RMSSD in condition 1. t2, differences between conditions 2 and 3, higher SDNN and RMSSD in condition 2.	3
(H. I. Jo et al. 2022); South Korea	60; mild symptoms of stress, anxiety and depression	Experiment; within	1. 9 different virtual environments, 3 urban, 3 waterfront, 3 green space, one of each category randomly selected; 1; 540s	Laboratory; HR, SDNN, LF, TP	**^b^** **Baseline (180s, t1), stress task (180s, t2), intervention (540s, t3):** t1→t2, increased HR in all three categories. t3, increased SDNN for waterfront and green space, not for urban environments.	3
(Jyskä et al. 2022); Finland	13; healthy	Experiment; within	1. Virtual environment with randomized loop of audiovisual stimuli of generative art and binaural audio; 1; 300s	Laboratory; HR, RMSSD, SDNN	**Pre‐intervention (> 300s, t1), intervention (300s, t2), post‐intervention (> 300s, t3):** t1→t3, increased SDNN and RMSSD, no changes in HR.	3
(Kamińska et al. 2020); Poland	28; healthy office workers	Crossover randomized experiment; within	1. Six EMDR exercises in virtual forest, moving golden sphere; 1; 632s 2. Six EMDR exercises in virtual forest, moving golden sphere and sound of a gong; 1; 623s 3. Six EMDR exercises in virtual forest, moving golden sphere, haptic feedback; 1; 623s 4. Six EMDR exercises in virtual forest, moving golden sphere, sound of a gong, and haptic feedback; 1; 623s	Laboratory; HR, GSR	**^d^** **Intervention:** HR decreased in all conditions, largest in conditions 2 and 3. GSR decreased in all conditions, largest in conditions 1 and 2.	3
(Kamińska et al. 2024); Poland	55; Refugees	Pilot study/experiment; within	1. Seven sets of exercises including breathing, visualisation, and BLS exercises, in virtual apartment with mountains; max 5; x	Laboratory; GSR	**Session:** GSR decreased within each session, most in the first session.	3
(Kazzi et al. 2018); Australia	Study 1: 16; healthy. Study 2: 16; healthy	Crossover randomized experiment; within	1. Abstract virtual environment with guided relaxation and breathing; 1/1; 300s/300s 2. 2D guided relaxation and breathing (only in study 1); 1; 300s 3. Seated rest; 1/1; 300s/300s	Laboratory; HR, SBP, DBP, LF, HF, LF/HF, TP	**Study 1: Intervention:** No differences in HR, SBP, DBP, LF, HF, LF/HF, TP between conditions. **Study 2: baseline (t1), stress task 1 (t2), stress task 2 (t3), intervention (t4):** t2→t3, no changes in HR, increased SBP, DBP, and LF, and decreased HF. Conditions 1, higher LF and lower HF than condition 3. t1→t4, no differences in HR and SBP.	3
(H. Kim and Jeon 2021); South Korea	83; healthy and highly stressed	Experiment; within	1. Virtual video of nature walk; 1; 630s	Laboratory; BVP, IBI, HRV total, RMSSD, SDNN, NN50, VLF, LF, HF, LF/HF, SC, RR	**^e^** **Baseline:** Positive correlation of RR and IBI with NRS, negative correlation of SDNN and RMSSD with NRS. RR is associated with increased STAI, RR and IBI are associated with increased NRS.	3
(H. Kim et al. 2021); South Korea	83; healthy and highly stressed	Open randomized crossover trial; mixed	1. Virtual video of nature walk; 1; 630s 2. BF, physiological signals displayed on a screen; 1; 630s	Laboratory; BVP, IBI, HRV total, RMSSD, SDNN, NN50, pNN50, VLF, LF, HF, LF/HF, SC	**^b^** **Stress task (210s, t1), intervention (210s, t2):** t1→t2, decreased BVP, IBI, HRV total, LF, HF, SC, and increased NN50 and pNN50 in condition 1. Condition 2, increased NN50, pNN50, VLF, and decreased BVP, IBI, RMSSD, SDNN, and SC. Differences between conditions in NN50, pNN50, LF, LF/HF.	3
(I. Kim et al. 2023); USA	64; healthy	RCE; between	1. Virtual drawing by making strokes and changing colour and drawing tools; 1; 300s 2. Virtual throwing; 1; 300s 3. Virtual nature; 1; 300s 4. Waiting in room; 1; 300s	Laboratory; HR, RMSSD	**Relaxation (60s, t1), post‐stress task (60s, t2), post‐intervention (60s, t3):** t2→t3, decreased RMSSD in condition 3, no differences in conditions 1, 2, and 4. t1→t3, no differences in HR and RMSSD in condition 1, decreased RMSSD in condition 3.	3
(Kluge et al. 2021); Australia	30; healthy	Pilot efficacy trial/experiment; within	1. VR BF controlled breathing; 3; 1500–1800s	Laboratory; RR, RR variability	**Baseline (180s), pre‐session (180s), post‐session (180s):** t2→t3, decreased RR and RR variability. Decreased RR and RR variability from sessions 1 to 2, 1 to 3, and 2 to 3.	3
(Knaust et al. 2022); Germany	102; healthy	Counter‐balanced, RCE; within	1. Virtual beach; 1; 300s 2. Beach via PC screen; 1; 300s 3. Sit quietly on chair; 1; 300s	Laboratory; HR, SCL	**Baseline (t1), stress task (t2), intervention (5 × 60s, t3):** Time effect on HR, no condition or time x condition effect. t2, higher HR and SCL than t1 and t3. Time and time x condition effect, larger decreased SCL in condition 1 compared to 3, condition 2 compared to 3. No differences between conditions 1 and 2.	3
(Kothgassner et al. 2019); Austria	56; students	RCE; between	1. Real waiting room, human social support; 1; 300s 2. Virtual empty waiting room, agent social support with pre‐programed answers, manipulated by experimenter; 1; 300s. 3. Virtual empty waiting room, avatar social support with pre‐programed answers; 1; 300s 4. Virtual empty waiting room, no social support; 1; 300s	Laboratory; HR	**Baseline (60s t1), intervention (60s, t2), post‐stress task (60s, t3), recovery (60s, t4):** t1, no difference in HR. t3, increased HR for conditions 2 and 4. t3, higher HR in conditions 2 and 4 compared to 1, no difference between conditions 2 and 3. t4, no difference in HR.	2
(Kuhne et al. 2023); Australia	82; healthy	Crossover randomized experiment; within	1. VR, six scenes, forest (acclimatization to delivery modality), climbing gym (neutral), pub (tense), outer space (calming), carpark (tense), beach (calming); 1; 480s 2. 2D, same six scenes; 1; 480s	Laboratory; HR, RR, GSR	**^b^** **Baseline (180s, t1), intervention (480s, t2):** t1, no differences in HR, GSR, and RR between conditions. Higher HR and GSR, and lower RR in condition 1 than 2. Higher HR tense scenes in condition 1. HR: exposure, scene, and exposure x scene effect. t1→t2, higher HR for neutral and tense scenes in condition 1. Higher GSR for tense scenes in condition 1. GSR: Exposure and scene effects, no exposure x scene, differences between forest, climbing, and pub scenes. GSR increased in the first 3 scenes and decreased in the last 3. RR: scene, no exposure or scene x exposure effects. Condition 1, lower RR in the climbing scene compared to condition 2.	3
(Lan et al. 2021); Taiwan	20; students	RCE; between	1. VR slow breathing exercise with feedback; 8; 1200s 2. VR slow breathing exercise without feedback; 8; 1200s	Laboratory; BR	**Baseline (120s, t1), session (1200s, t2)**: t1, no difference in average BR between conditions. t1→t2, differences in average BR between conditions, in all sessions except the first, lower BR in condition 1.	3
(Liszio et al. 2018); Germany	62; healthy	RCE; between	1. VR, underwater simulation; 1; 420s 2. Screen, underwater simulation; 1; 420s 3. No distraction; 1; 420s	Laboratory; SDSD, cortisol	**Baseline (t1), stress task (1200s, t2), intervention (420s, t3):** Time and time x condition effects on SDSD, no condition effect. t3, highest SDSD for condition 1. Differences between conditions 1 and 2, and conditions 1 and 3. **Baseline (t1), stress task + 15 min (t2), post‐intervention (t3):** Time effect on cortisol, no condition or time x condition effect. t2→t3, decrease cortisol, stronger decrease conditions 1 and 2 compared to 3. No differences between all conditions.	3
(Liszio and Masuch 2019); Germany	57; healthy	RCE; between	1. Interactive VR, animated virtual beach with mini games; 1; 540s 2. Non‐interactive VR, animated virtual beach; 1; 540s 3. Waiting; 1; 540s	Laboratory; SDSD	**Baseline (t1), intervention (540s, t2), post‐intervention (t3), stress task (t4):** Time effect, higher SDSD at t2 in condition 1 compared to 2, no difference at t4. t1→t2, difference between three conditions, highest increased SDSD in condition 1, decreased SDSD in conditions 2 and 3 that differed significantly. t3→t4, no changes in all conditions.	3
(Liu et al. 2023); USA	31; veterans	Experiment; within	1. Virtual guided meditation, option between 6 different virtual scenes (mountain, forest, beach, desert, lake, cave); 1; 600s	Laboratory; HR, SBP, DBP	**Pre‐intervention (t1), post‐intervention (t2):** t1→t2, decreased SBP, DBP, and HR	3
(Lopes et al. 2022); Canada	16; healthy	Counter‐balanced experiment; within	^f^1. Video only, nature walk; 1; 75s 2. AV nature walk; 1; 75s 3. AV + olfactory nature walk; 1; 75s. 4. AV + olfactory somatosensory, vibroacoustic nature walk; 1; 75s	^g^Laboratory; BVP, M + SD of negative 1st diff. EDA, LF, HF	**^b^** **Intervention:** Difference between conditions 2 and 4 in M and SD of negative 1st difference of EDA signal	3
(Ma et al. 2021); China	39; students	Experiment; within	1. Four virtual scenes (lakeside, beach, lake, underwater). In fixed order; 1; 1200–1440s 2. Simulated medical environment; 1; 300–360s 3. Self‐measurement; 1; 300–360s	Laboratory; pulse, SBP, DBP	**Intervention:** No difference in SBP, DBP and pulse between 4 scenes in condition 1. In condition 1, higher SBP and DBP than 2 and 3. No difference in pulse between conditions 2 and 3. Higher pulse condition 2 than first scene condition 1.	3
(Manaf et al. 2021); Malaysia	18; healthy	Randomized experiment; mixed	1. Interactive virtual forest; 1; 300s 2. Forest environment with interaction on desktop; 1; 300s	Laboratory; HR, SC	^h^ **Baseline (60s, t1), stress task (60s, t2), intervention (60s, t3):** t2, increased HR and SC. t3 decreased HR and SC at timepoint 3.	3
(Mazgelytė et al. 2021); Lithuania	43; healthy	Crossover experiment; within	1. EEG VR BF, tropical island, weather shifts with alpha percentage; 1; 720s 2. Mindfulness‐based VR BF, blue dot moves with head movement, changes objects; 1; 720s 3. VR RBF, night sky, red/blue lines for heart and breathing rates; 1; 720s 4. VR GSR BF, tropical island, weather changes with arousal; 1; 720s	Laboratory; HR, GSR, cortisol	**First‐minute intervention (t1), last‐minute intervention (t2):** t1→t2, increased GSR in all conditions, largest in condition 3. No change in HR in all conditions. **Pre‐intervention (t1), post‐intervention (t2):** t1→t2, conditions 1, 2, and 4, decreased cortisol, largest in condition 2.	3
(Mazgelytė et al. 2022); Lithuania	39; healthy	Experiment; within	1. VR‐based RBF‐assisted relaxation exercise; 1; 720s	Laboratory; HR, RMSSD, pNN50, GSR, RR, cortisol	**Pre‐intervention (t1), intervention (t2), post‐intervention (t3):** t1→t3, decreased HR, RR, and increased GSR. No changes in RMSSD and pNN50. **Pre‐intervention (t1), post‐intervention (t2):** t1→t2, decreased cortisol	3
(McGarry et al. 2023); Australia	21; healthy	Experiment; within	1. VR beach environment; 1; 900s	Laboratory; HR	**Pre‐intervention (t1), post‐intervention (t2):** t1→t2, decreased HR.	3
(Mostajeran 2021); Germany	35; healthy	Counter‐ balanced experiment; within	1. Combinations of: immersion (video or slideshow), environment (forest or urban); 4; 360s	Laboratory; HR, GSR	**Baseline (360s, t1), stress task (300s, t2), session (360s, t3):** Time effect on HR, lowest at t3, no difference t1→t2. Time and immersion x time effects on GSR. t2, higher GSR. t2→t3, no effects on HR. Immersion effect, larger decrease GSR slideshow than video. Larger decrease GSR urban slideshow and forest slideshow than urban video.	3
(Naef et al. 2022); Switzerland	42; healthy	Counter‐balanced controlled experiment; within	1. Audiovisual: 42 virtual videos of Switzerland with sound; 1; 1800s 2. Visual: 42 virtual videos of Switzerland, no sound; 1; 1800s 3. Auditory: Nature sounds; 1; 1800s 4. No video or sound; 1; 1800s	Laboratory; HR, BP, RR	**Intervention:** Lower HR in condition 1 than 3 and 4. Higher RR in condition 1 than 2, 3, and 4. Higher BP in conditions 2, 3, and 4 than in 1. Condition x duration effect on HR in conditions 3 and 4 and RR in conditions 1 and 4.	3
(Naylor et al. 2019); Australia	49; non‐clinical	RCE; mixed	1. Interactive meditation, changing visuals based on input; 1; 1200s 2. Guided breathing exercise with generated visuals; 1; 1200s 3. Rainy day office window; 1; 1200s	Laboratory; HR	**Stress task (t1), intervention (4 × 300s, t2), post‐intervention (t3):** Time effect on HR, no condition or condition x time effect. Decrease HR from t1→t2 and t1→t3, no difference t2→ t3.	3
(Pallavicini et al. 2009); Italy	13; GAD	RCT; between	1. Virtual tropical island, relaxation‐focused exposure techniques, HR BF; 8; x 2. Virtual tropical island, relaxation‐focused exposure techniques; 8; x 3. Waiting list; 4; x	Laboratory; HR, GSR	**Pre‐session (180s, t1), post‐session (180s, t2):** t1→t2, no changes in HR and GSR for both conditions.	3
(Pardini et al. 2024); Italy	72; students	Pilot RCT; between	^i^1. Passive PMRT with personalised VR exposure (720s); 1; 720s 2. PMRT and GI by a psychotherapist; 1; 720s	Laboratory; HR frequency	**Baseline (t1), pre‐intervention (5 × 60s, t2), intervention (12 × 60s, t3), post‐intervention (5 × 60s, t4):** Condition effect on HR frequency, no time effect. t3→t4, time x condition effect on HR, decreased HR for condition 1 compared to 2.	2
(Park et al. 2020); UK	32; healthy	Experiment; within	1. Scenes based on combinations of: auditory forms (audio‐visual or no audio), visual forms (VR or screen), scenes (rural without water (only VR), rural with water or urban; 10; 60s	Laboratory; HR, EDA, RR	**Baseline (60s, t1), stress task (60s, t2), session (60s, t3):** t2, increased HR. Auditory forms effect on RR, not HR and EDA. No visual form effect on HR, RR, and EDA. Scenes' effect on HR and RR, not EDA. No auditory x visual forms and visual x scene effects on HR, RR, and EDA. Auditory x scenes effect on HR, not RR and EDA. No auditory x visual x scene effect on HR, RR, and EDA.	3
(Pascual et al. 2023); USA	45; healthcare workers	RCE; between	1. VR guided meditation; x; x 2. Mobile guided meditation; x; x	Ambulatory; LF	**Session:** No difference in average LF between conditions. Intrasession LF change was larger in condition 1 than 2.	3
(Richesin et al. 2021); USA	44; students	RCE; within	1. 3D VR artmaking and design; 1; 900s 2. Traditional artmaking with markers and pencils; 1; 900s 3. Virtual simulation to familiarise with VR controls; 1; 900s	Laboratory; HR, SC	**Pre‐intervention (300s, t1), post‐intervention (300s, t2):** t1→t2, decreased HR for all conditions, no difference in SC. Condition x time effect on HR. Greater decrease HR for condition 1 than condition 3.	3
(Rockstroh et al. 2019); Germany	68; healthy	RCE; mixed	1. VR HRV‐BF, virtual beach environment; 1; 600s 2. Traditional HRV‐BF with colourful dots; 1; 600s 3. Neutral nature video; 1; 600s	Laboratory; HR, RMSSD, SDNN, LF/HF	**Pre‐intervention (t1), mid‐intervention (t2), post‐intervention (t3):** HR and RMSSD, both time effects, no condition or time x condition effects. t1→t2, no difference in HR, t2→t3 decrease HR. t1→t2, increased RMSSD, t2→t3 decreased RMSSD. Condition and time x condition effects on SDNN and LF/HF. t2, higher SDNN and LF/HF in conditions 1 and 2 compared to 3, no difference between conditions 1 and 2.	2
(Rockstroh et al. 2020); Germany	94; healthy	Randomized experiment; between	1. Virtual forest with BF; 1; 600s 2. Virtual forest, no BF; 1; 600s 3. Forest on screen with BF; 1; 600s 4. Forest on screen, no BF; 1; 600s	Laboratory; EDA	**Intervention (10 × 60s):** Start of intervention, no difference in EDA between conditions. Time effect on EDA. Minutes 1→5, EDA decreased stepwise. Smaller decrease from minutes 5→10. No display type, BF, display type x BF, display type x time, time x BF, and display type x BF x time effects on EDA.	3
(Sun et al. 2023); USA	63; pregnant	Randomized experiment; between	1. Virtual urban park with 50% green; 1; 300s 2. Virtual urban stress view with 12% green; 1; 300s 3. Virtual urban street view without green; 1; 300s	Laboratory; HR, SBP, DBP, SCL, cortisol	**Baseline (300s, t1), stress task (900s, t2), intervention (300s, t3):** t1→t2, increase HR and SCL. t2→t3, no difference in HR and SCL between conditions. **Baseline (t1), post‐stress task (t2), post‐intervention (t3):** t2, increase SBP. t2→t3, decreased SBP in condition 3 compared to 1 and 2. Not differences in DBP. **Baseline (t1), post‐stress task (t2), post‐intervention (t3), post‐intervention + 15 min (t4), post‐intervention + 30 min (t5):** Increased cortisol t1→t2. No differences in cortisol at t3‐t5 between conditions.	3
(Tan et al. 2021); Singapore	41; psychiatric patients	Pilot RCT; between	1. Virtual exercises of abdominal breathing, muscle relaxation, GI, and relaxing content; 2; 1200s 2. Sitting and reading health promotion pamphlets; 2; 2400s	Laboratory; HR, SBP, DBP	**First‐minute session (t1), last‐minute session (t2):** No changes in SBP, DBP, and HR for both conditions	3
(Tinga et al. 2019); Netherlands	60; students	RCE; between	1. VR meditation with RB, moving cloud based on respiration; 1; 348s 2. VR meditation with automatic movement of white cloud; 1; 348s 3. VR meditation without feedback, only a blue background; 1; 348s	Laboratory; HR, RMSSD	**Baseline (60s, t1), stress task (300s, t2), intervention (348s, t3):** t1→t2, increased HR, no change in RMSSD. t2→t3, larger decrease HR in condition 1 than 2. No differences between conditions 1 and 3 in HR and RMSSD, and no difference in RMSSD between conditions 1 and 2.	3
(Valtchanov et al. 2010); Canada	22; students	RCE; mixed	1. Virtual nature environments; 1; 600s 2. Slideshow of abstract paintings; 1; 600s	Laboratory; IBI, SCL	**Baseline (120s, t1), post‐stress task (120s, t2), post‐intervention (120s, t3):** t1→t2, decreased IBI and increased SCL. At t2, no difference in SCL and IBI between conditions. Time x condition effect on SCL, decreased SCL from t2→t3 for condition 1, not 2. No time x condition effect on IBI.	3
(Villani and Riva 2008); Italy	60; students	RCE; mixed	1. Virtual forest with relaxation exercises; 2; x 2. DVD relaxation exercises; 2; x 3. Audio relaxation exercises; 2; x 4. No treatment; 2; x	Laboratory; HR, SC, RR	**Pre‐session (t1), post‐session (t2):** Condition 1, decreased RR in both sessions. Condition 2, decreased HR and increased SC in both sessions. Condition 3, decreased HR in both sessions, increased SC in session 1.	3
(Villani and Riva 2012); Italy	36; healthy	RCE; mixed	^i^1. Virtual nature environments with relaxation exercises; 3; x 2. Video of nature environments with relaxation exercises; 3; x 3. Audio of relaxation exercises; 3; x	Laboratory; HR	**Baseline (t1), pre‐session (t2), post‐session (t3):** t2→t3, time and time x condition effects on HR, decreased HR in all conditions, largest in condition 1.	3
(Voigt‐Antons et al. 2021); Germany	27; healthy	RCE; within	1. Virtual forest with search task, high interaction; 1; 300s 2. Virtual forest to explore, low interaction; 1; 300s 3. Virtual forest; 1; 300s 4. Virtual gray environment with white cross; 1; 300s	Laboratory; BPM, RMSSD	**Stress task (60s, t1), intervention (last 60s, t2):** t2 compared to t1, lower BPM and higher RMSSD, all conditions 1–3 combined.	3
(Weibel et al. 2023); Switzerland	107; healthy	Randomized experiment; mixed	1. HRV‐BF, virtual mountains; 2; 600s 2. HRV‐BF on screen, mountainous landscape; 2; 600s 3. sPB, virtual mountains; 2; 600s 4. sBP on screen, mountainous landscape; 2; 600s	Laboratory; HR, SDNN, LF	**^b^** **Baseline (t1), session 1 (t2.1), session 2 (t2.2), post‐session (t3):** t1→t3, time effect on HR, decreased HR in all conditions. Time effect on SDNN, t1→t2.1 increased SDNN, t2.2→t3 decreased SDNN. Time, technology, and technique x time effects on LF, t1→t2.1 increased LF, t2.2→t3 decreased LF, higher at t3 than t1.	2
(Yin et al. 2018); USA	31; healthy	Crossover randomized experiment; within	1. Virtual classroom; 1; 300s 2. Physical classroom; 1; 300s 3. Virtual office common room; 1; 300s 4. Physical office common room; 1; 300s	Laboratory; HR, SBP, DBP, SCL	**Baseline (t1), session (t2):** No differences in HR. t1→t2, decreased SCL in condition 1. **Baseline (t1), post‐session (t2):** t1→t2, decreased SBP in conditions 1 and 2, increased SBP in conditions 3 and 4. Differences between conditions 1 and 3, 2 and 4. Decreased DBP in condition 1, increased in condition 3. Differences in DBP between conditions 1 and 3.	3
(Yin et al. 2020); USA	100; healthy	RCE; between	1. Virtual office with indoor green and living wall; 1; 360s 2. Virtual office with outdoor view of trees and grass; 1; 360s 3. Virtual office with indoor green and outdoor view; 1; 360s 4. Virtual office, no green; 1; 360s	Laboratory; HR, SBP, DBP, RMSSD, LF/HF, SCL	**Baseline (300s, t1), stress task (420s, t2), intervention (360s, t3):** At t1, no differences in log RMSSD, log LF/HF, HR, and log SCL. t1→t2, increased HR, LF/HF, and SCL, no change in RMSSD. t3, faster increase RMSSD conditions 1, 2, and 3, especially condition 1, no changes in SCL, HR, LF/HF. **Baseline (t1), post‐stress task (t2), post‐intervention (t3):** t1, no differences in SBP and DBP. t1→t2, increased DBP. Compared to condition 4, larger decrease in SBP and DBP in other conditions, most in condition 1.	3
(Yu et al. 2020); Taiwan	34; healthy	Crossover RCE; within	1. Virtual forest; 1; 600s 2. Virtual urban setting; 1; 600s	Laboratory; HR, SBP, DBP, LF, HF, LF/HF	**Pre‐intervention (360s, t1), post‐intervention (360s, t2):** Time effect on HR, no condition or time x condition effects. t1→t2, decreased HR in both conditions. No time, conditions, or time x condition effect on SBP, DBP, LF, HF, LF/HF.	3
(Yuan et al. 2022); China	63; elderly	RCE; mixed	1. Virtual forests; 3; 300s 2. Continue usual routines; 3; 300s	Ambulatory; SBP, DBP	**Day 1: Pre‐session day 1, post‐session day 1:** No time, condition, time, or time x condition effects on SBP and DBP **Day 1, pre‐session 1 (t1), post‐session 1 (t2). Day 2 pre‐session 2 (t3), post‐session 2 (t4). Day 3 pre‐session 3 (t5), post‐session 3 (t6):** Time effect on SBP and DBP. t1→t2 and t1→t3 condition 1, no changes. t1→t4 and t1→t6, decreased SBP and DBP, no time x condition effect.	3

Abbreviations: ApEN, Approximate entropy; app. RR, approximate respiration rate; AV, audiovisual, BF, biofeedback; BP, blood pressure; BPM, beats per minute; BR, breathing rate; BVP, blood volume pulse; DB, diaphragmatic breathing; DBP, diastolic blood pressure; EDA, electrodermal activity; EMDR, Eye Movement Desensitisation and Reprocessing; GAD, generalised anxiety disorder; GI, guided imagery; GSR M, galvanic skin response mean; GSR SD, galvanic skin response standard deviation; HF, high frequency (in 0.15–0.4 Hz range); HR, heart rate; HR M, heart rate mean; HR max, heart rate max; IBI, interbeat interval; LF, low frequency (in 0.04–0.15 Hz range); LF/HF, ratio of low frequency to high frequency; MD, mead difference; nHF, normalised high frequency power; nLF, normalised low frequency power; NRS, numeric rating scale; PMR, progressive muscle relaxation; PMRT, progressive muscle relaxation technique; RB, respiratory biofeedback; RCE, randomized controlled experiment; RCT, randomized controlled trial; RMSSD, root mean square of the successive differences; RR, respiration rate; SBP, systolic blood pressure; SC, skin conductance; SCL, skin conductance level; SD 1, standard deviation of Poincaré plot perpendicular to the line‐of‐identity; SD 2 standard deviation of the Poincaré plot along the line‐of‐identity; SDNN, standard deviation of the NN (R‐R) intervals; sPB, standardized paced breathing; STAI‐X, State‐Trait Anxiety Inventory; TP, total power; VR, virtual reality.

^a^ Machine learning‐based classification and signal processing techniques.

^b^ Only significant results (*p* < 0.05) are reported in the table.

^c^ Five assimilar binary classifiers to determine stress and relaxation conditions.

^d^ Descriptive statistics, difference between the mean value of the first break scene and the mean of the closing break (method 1), and the difference mean value between the third minute and the mean value of 15th minute (method 2).

^e^ Only correlations with STAI‐X and NRS are reported.

^f^ Only conditions 2 and 4 are included in the analysis.

^g^ More physiological parameters are mentioned.

^h^ Percentage change calculations.

^i^ Part of a larger intervention.

### 3.2 Types of VR Stress Interventions

Most studies (*n* = 61) specified measuring the effects of VR systems on relaxation or stress reduction, while the remaining eight focused on investigating the physiological effects of the VR system. Fifty‐two studies used a form of virtual natural environments or elements, 43 of which were conducted in the general population setting. Beyond nature exposure, 10 studies incorporated biofeedback in the VR system (Aganov et al. 2020; Blum et al. 2019, 2020; Kluge et al. 2021; Mazgelytė et al. 2021, 2022; Pallavicini et al. 2009; Rockstroh et al. 2019, 2020; Weibel et al. 2023), five included relaxation or meditation exercises (Chavez et al. 2020; Kamińska et al. 2024; Liu et al. 2023; Villani and Riva 2008, 2012), and one included biofeedback and meditation (Tinga et al. 2019). All 69 studies focused on short‐term effects, defined as outcomes assessed before and after VR use or during its use. Only one study investigated long‐term effects, with a follow‐up at 3 months (De Jesus Junior et al. 2023). The VR sessions varied between 60 and 1800 s, with a median duration of 600 s. The included studies demonstrated considerable heterogeneity in intervention content and dose. Most interventions involved virtual nature exposure, ranging from brief single‐session experiences to multi‐component protocols incorporating biofeedback or meditation.

#### 3.2.1 Types of Stressors

Among the 69 selected studies, 26 (37.7%) incorporated a stress task before or after the VR interventions (see Table 1). Stressors varied in type and intensity, and can be broadly categorised into cognitive, social‐evaluative, and emotional stressors. The most common were cognitive stressors, used in 16 studies, including mental arithmetic tasks, such as adding three‐digit numbers (Aganov et al. 2020; Anderson et al. 2017), serial subtraction (H. I. Jo et al. 2022), summing random numbers (Jin et al. 2023), the Stroop task which requires colour‐word conflict resolution (Blum et al. 2019; Naylor et al. 2019), the Letter Detection Test, involving rapid visual scanning (Manaf et al. 2021), and the digit span task with reverse recall and summation (Chen and Li 2022). Social‐evaluative stressors were applied in seven studies using the Trier Social Stress Test (TSST), which combines public speaking and mental arithmetic under evaluation (I. Kim et al. 2023; Kothgassner et al. 2019; Liszio et al. 2018; Liszio and Masuch 2019; Mostajeran 2021; Sun et al. 2023; Tinga et al. 2019). Emotional stressors included horror videos (Park et al. 2020; Voigt‐Antons et al. 2021), roller coaster simulations (Gu et al. 2021), and reflective tasks such as recalling academic stress (Gonzalez et al. 2021). The diversity in stressor type contributes to the heterogeneity in study designs and may influence the observed effects in VR stress interventions.

#### 3.2.2 Ambulatory and Laboratory Studies

Regarding the study setting, most studies (*n* = 63) conducted physiological assessments in a laboratory setting, offering controlled conditions conducive to reliable data collection. In contrast, five studies were conducted in ambulatory settings, where physiological responses were measured using wearable or portable devices during participants' use of VR interventions in their natural environments (e.g., home, workplace) rather than controlled laboratory settings. Of these, three took place in a professional context. One study was conducted in both laboratory and ambulatory settings, allowing for direct comparison across settings. Thus, study settings varied considerably, with most favoring laboratory control, underscoring heterogeneity in ecological validity across the evidence base.

### 3.3 Theoretical Frameworks for Selecting Physiological Measures

The selected studies utilised various frameworks to guide the selection of physiological stress markers. A majority (56; 81%) provided a hypothesis or rationale linking physiological outcomes to relaxation‐related outcomes. A common rationale was the expectation of reduced autonomic arousal, reflected in markers such as decreased HR (Gonzalez et al. 2021), decreased GSR (Kamińska et al. 2024), and increased SDNN and LF/HF (Ho et al. 2023). These patterns were most consistently observed in studies targeting the general population (50/59; 85%). In contrast, studies conducted in mental healthcare (1/4; 25%) and professional settings (5/6; 83%) showed less emphasis on physiological measures overall.

A total of 19 studies explicitly defined the concept of stress response or stressor. Four studies, primarily in the general population settings, referenced Selye's (1956) foundational stress theory (Gonzalez et al. 2021; Kamińska et al. 2020; Mazgelytė et al. 2022; Naef et al. 2022). Two studies conducted in the professional settings focused on work‐related stress (Adhyaru and Kemp 2022; Ho et al. 2023). The remaining 13 studies offered definitions related to stress response, stressor or relaxation, drawing on a range of conceptual frameworks (Alyan et al. 2021; Ishaque et al. 2020; Jin et al. 2023; Kamińska et al. 2024; H. Kim et al. 2021; I. Kim et al. 2023; Kluge et al. 2021; Kothgassner et al. 2019; Manaf et al. 2021; Mazgelytė et al. 2021; McGarry et al. 2023; Tan et al. 2021; Villani and Riva 2012). These findings suggest that only a minority of studies explicitly define stress‐related constructs, while those that do tend to align with established theoretical models, highlighting ta need for greater conceptual clarity and consistency across research contexts.

As a supportive theory of beneficial exposure to virtual nature, 25 studies (36.2%) cited Attention Restoration Theory (ART) (Adhyaru and Kemp 2022; Anderson et al. 2017; Blum et al. 2019; Calogiuri et al. 2018; Cawley and Tejeiro 2024; Chan et al. 2023; Frigione et al. 2022; Gerber et al. 2019; S.‐H. Jo et al. 2021; Kamińska et al. 2024; Knaust et al. 2022; Lan et al. 2021; Liszio et al. 2018; Ma et al. 2021; Mostajeran 2021; Park et al. 2020; Rockstroh et al. 2019, 2020; Sun et al. 2023; Valtchanov et al. 2010; Voigt‐Antons et al. 2021; Yin et al. 2018, 2020; Yu et al. 2020; Yuan et al. 2022). Of these, 23 were conducted in the general population and two in the professional setting. Twenty‐two studies (31.9%) mentioned the Stress Recovery Theory (SRT) or cited one of Ulrich's foundational works on stress recovery (1983; 1991), though not all explicitly named the theory. These studies were all in the general population setting (Alyan et al. 2021; Anderson et al. 2017; Chan et al. 2023; Frigione et al. 2022; Gerber et al. 2019; Gu et al. 2021; Jin et al. 2023; S.‐H. Jo et al. 2021; Kamińska et al. 2024; Knaust et al. 2022; Mostajeran 2021; Naef et al. 2022; Park et al. 2020; Rockstroh et al. 2019, 2020; Sun et al. 2023; Valtchanov et al. 2010; Voigt‐Antons et al. 2021; Yin et al. 2018, 2020; Yu et al. 2020; Yuan et al. 2022). Three studies in the general population setting mentioned the biophilic hypothesis (Chan et al. 2023; Sun et al. 2023; Yin et al. 2020), which posits an innate human affinity for nature (Wilson 1984), and two studies the Psycho‐Evolutionary Theory (S.‐H. Jo et al. 2021; Sun et al. 2023), which links nature exposure to reduced sympathetic nervous system (SNS) activation and emotional stress (Ulrich 1984). These findings indicate that ART and SRT are the predominant theoretical models guiding research on VR stress research, while alternative frameworks remain underutilised, suggesting opportunities for broader theoretical integration in future studies.

#### 3.3.1 Psychological Measures

Most studies investigated both changes in physiological markers and psychological changes. However, two general population studies focused more on the qualitative findings, with limited reporting of physiological data (H. Kim and Jeon 2021; McGarry et al. 2023). While this multimodal approach reflects the multidimensional nature of stress, studies rarely clarified the theoretical rationale for including both measurement types or examined how they relate to one another. The most common self‐reported measures were the Perceived Stress Scale (PSS), Positive and Negative Affect Schedule (PANAS), and State‐Trait Anxiety Inventory (STAI).

While 16 studies administered the PSS, only three explicitly examined correlations with physiological markers, and all found no significant relationship (Jyskä et al. 2022; H. Kim and Jeon 2021; Richesin et al. 2021). Eight studies used the PSS solely for baseline sample characterisation rather than as an outcome measure (H. I. Jo et al. 2022; H. Kim et al. 2021; H. Kim and Jeon 2021; I. Kim et al. 2023; Mazgelytė et al. 2021, 2022; Sun et al. 2023; Weibel et al. 2023). Among studies reporting PSS outcomes, several found significant effects (El‐Qirem et al. 2023; Richesin et al. 2021), while others found no intervention effects (Cawley and Tejeiro 2024; Ho et al. 2023; Jeong et al. 2022; Mostajeran 2021). One study investigated the correlation between PSS and school stress and substance use (Crosswell and Yun 2022).

Fourteen studies employed the PANAS, which measures emotional states such as excitement or hostility (Watson et al. 1988). Three used PANAS only for baseline characterisation (H. Kim et al. 2021; H. Kim and Jeon 2021; I. Kim et al. 2023). Among the remaining 11 studies, four studies found the expected increase in positive affect and decrease in negative affect (Anderson et al. 2017; Liszio et al. 2018; Sun et al. 2023; Yuan et al. 2022), three found decreased negative affect only (Chan et al. 2023; Gu et al. 2021; Richesin et al. 2021), one found a decrease in both positive and negative affect (Naylor et al. 2019), one only an increase in positive affect (Ho et al. 2023), and two found no significant changes (Liszio and Masuch 2019; Villani and Riva 2008). Only one study examined the relationship between PANAS and physiological measures, finding no significant relationships (Richesin et al. 2021).

Seventeen studies used the STAI, which distinguishes between temporary and long‐term (trait) anxiety (Spielberger et al. 1983). Five studies used STAI for baseline characterisation (H. I. Jo et al. 2022; H. Kim et al. 2021; H. Kim and Jeon 2021; Mazgelytė et al. 2021, 2022). While 11 reported significant anxiety reductions (Aganov et al. 2020; Chavez et al. 2020; Liszio et al. 2018; Liszio and Masuch 2019; Pallavicini et al. 2009; Pardini et al. 2023; Richesin et al. 2021; Rockstroh et al. 2020; Villani and Riva 2008, 2012; Yin et al. 2020), only two examined relationships with physiological measures, both finding no associations (H. Kim and Jeon 2021; Richesin et al. 2021). While most studies employed both physiological and psychological measures, few studies examined their relationship.

### 3.4 Physiological Markers and Outcomes

#### 3.4.1 Heart Rate

Fifty‐five studies assessed a form of heart rate (HR), 47 in the general population setting, four in the mental healthcare setting, and five in the professional setting. The most commonly reported physiological outcome was HR (*n* = 46). Other forms included HR mean (HR M; *n* = 1), HR max (*n* = 1), HR frequency (*n* = 1), interbeat interval (IBI; *n* = 5), blood volume pulse (BVP; *n* = 3), and pulse (*n* = 2). Across all forms of HR and settings, 29 studies measured a decrease in HR, with 21 of these using virtual natural environments, suggesting a potential calming effect of nature‐based VR content. Twenty‐two studies used a stress task, of which 12 studies finding increased HR caused by a stress task. In contrast, 16 studies did not report any changes in HR.

Effect sizes for HR and BPM were reported in 21 studies, ranging from small to very large. These were predominantly conducted in the general population setting (Alyan et al. 2021; Cawley and Tejeiro 2024; Chan et al. 2023; Ho et al. 2023; Knaust et al. 2022; Kothgassner et al. 2019; Kuhne et al. 2023; Liu et al. 2023; Mazgelytė et al. 2022; McGarry et al. 2023; Mostajeran 2021; Naylor et al. 2019; Park et al. 2020; Richesin et al. 2021; Rockstroh et al. 2019; Tinga et al. 2019; Valtchanov et al. 2010; Voigt‐Antons et al. 2021; Weibel et al. 2023; Yu et al. 2020), with one study in the professional setting (Tan et al. 2021). VR stress interventions, mostly nature‐based environments, generally reduce HR though the magnitude of effects varies considerably across study designs and populations.

#### 3.4.2 Heart Rate Variability

Twenty‐nine studies measured a form of HRV; 25 of these studies were in the general population, three in the professional setting, and one in the mental healthcare setting. In the time domain, RMSSD (*n* = 17), SDNN (*n* = 12), NN50 (*n* = 2), pNN50 (*n* = 3), and SDSD (*n* = 2) were assessed. Among RMSSD studies, 11 used virtual natural environments, and seven included a stress task. Seven studies reported increased RMSSD, while five found no change. Effect sizes for RMSSD were reported in three studies, of whichtwo reported small effect sizes and one exceptionally large effect (Chan et al. 2023; Tinga et al. 2019; Voigt‐Antons et al. 2021). SDNN increased in seven studies, five of which used virtual natural environments without stress tasks. Three studies reported no significant SDNN changes. SDNN effect sizes, reported in two studies, ranged from small to very large (Ho et al. 2023; Weibel et al. 2023).

In the frequency domain, 19 studies measured nLF, (*n* = 1), nHF, (*n* = 1), VLF (*n* = 3), LF (*n* = 14), HF (*n* = 12), LF/HF ratio (*n* = 13), and TP (*n* = 4). Sixteen were in the general population and three in the professional setting. Nine studies measuring LF used virtual natural environments, four used a stress task, and five reported increased LF. Four studies found a decrease in LF. Eight studies measured HF using virtual natural environments, and four used a stress task. Five studies found an increase in HF, and two found a decrease. Twelve studies measured LF/HF using virtual natural environments, and seven used a stress task. Six studies found no changes in LF, four found no changes in HF, and five found no changes in LF/HF. LF effect sizes ranged from small to very large (Ho et al. 2023; Weibel et al. 2023; Yu et al. 2020), and for HF and LF/HF, only small effects (Ho et al. 2023; Yu et al. 2020). These results show that VR stress interventions generally promote modest improvements in HRV, particularly in time‐domain measures, though effects vary widely across study designs and marker types.

#### 3.4.3 Electrodermal Activity

Twenty‐six studies measured electrodermal activity, reporting a wide range of parameters, including electrodermal activity (EDA; *n* = 5), skin conductance (SC; *n* = 5), skin conductance level (SCL; *n* = 8), and galvanic skin response (GSR; *n* = 8). Twenty‐three studies in the general population setting, two in the professional setting, and one in the mental healthcare setting. Fifteen studies found a decrease in EDA, SC, SCL, or GSR, of which 11 used virtual natural environments, and eight incorporated a stress task. Three studies reported no changes. The effect sizes for SCL, GSR, EDA, and SC were reported in nine studies in the general population setting, ranging from small to large, with one extreme value (Alyan et al. 2021; Knaust et al. 2022; Kuhne et al. 2023; Mazgelytė et al. 2022; McGarry et al. 2023; Park et al. 2020; Richesin et al. 2021; Rockstroh et al. 2020). These results show that VR interventions yield inconsistent effects on electrodermal activity, though reductions are somewhat more frequent in studies using natural environments.

#### 3.4.4 Blood Pressure

Fifteen studies used either systolic blood pressure (SBP; *n* = 13) and diastolic blood pressure (DBP; *n* = 13) or mean blood pressure (BP: *n* = 2), of which 13 were in the general population setting. Nine studies used virtual natural environments, in which one used a stress task, and three studies found a decrease in BP. Of the 15 studies, five used a stress task, and three studies reported increased SBP or DBP caused by a stress task. In total, seven found a reduction in SBP, six found a reduction in DBP, one found a reduced BP, and five studies found no changes. Effect sizes ranged from small to moderate to large, with two studies reporting moderate effects in the general setting (Liu et al. 2023; Yu et al. 2020), one study in the mental healthcare setting (Tan et al. 2021), and one study in the professional setting (Ho et al. 2023). These results show that VR interventions produce mixed effects on blood pressure, with modest reductions most common but inconsistent across study design and populations.

#### 3.4.5 Cortisol

Five studies in general population settings measured cortisol at specific times. Four studies reported decreased cortisol levels post‐VR, three studies used virtual natural environments, and two studies used a stress task. Two studies reported the timing of assessments (Mazgelytė et al. 2021; Sun et al. 2023), with one explicitly controlling for diurnal variation in physiological stress markers by standardising data collection times (Sun et al. 2023). Three studies controlled for food intake before assessment (Mazgelytė et al. 2021, 2022; Sun et al. 2023). Effect sizes ranged from small to moderate to large (Chavez et al. 2020; Liszio et al. 2018; Mazgelytė et al. 2022). VR stress interventions may reduce cortisol levels, though the limited number of studies and insufficient standardisation of data collection times and control procedures constrain firm conclusions.

#### 3.4.6 Respiration Rate

A total of 13 studies in the general population setting were identified that measured respiration rate (RR; *n* = 11), breathing rate (*n* = 1), approximate respiratory rate (*n* = 1), and respiratory rate variability (*n* = 1). Of these, six studies reported a post‐VR reduction in RR, suggesting a calming effect. Notably, two of these six studies employed virtual natural environments and none involved a stress task. Effect sizes across these six studies ranged from small to very large (Kuhne et al. 2023; Mazgelytė et al. 2022; Park et al. 2020).

### 3.5 Interpretation and Decision Rules

Overall, the effects of VR stress interventions were strongest in laboratory‐based studies conducted in the general population context using single‐session virtual natural environments. Twenty‐four studies reported effect sizes (e.g., Cohen's d, *r,* or eta‐squared) to interpret the changes in physiological markers. In the general population, 22 studies reported effect sizes, with 17 concluding that their VR stress intervention effectively reduced stress based on changes in one or more physiological markers (e.g., Alyan et al. 2021; Knaust et al. 2022; Liszio et al. 2018).

Specifically, six studies conducted in ambulatory settings showed reductions in HR from pre‐to post‐intervention, suggesting a relaxing effect. However, effects on BP and HRV parameters were more variable. Compared to ambulatory studies, laboratory‐based studies provided more consistent evidence of VR stress intervention effects on more physiological markers. Four studies carried out in mental healthcare settings with clinical populations reported no or minimal effects. Although the subset of studies employed multi‐session designs or variations of the VR system, their outcomes were largely consistent with those of single‐use interventions. Most studies reported reductions in HR, while findings for other physiological markers varied. However, the substantial variability in study designs limits the ability to draw firm conclusions. Non‐nature VR systems elicit limited physiological evidence of relaxation. The few effects suggest that non‐nature environments can promote mild parasympathetic activation, but not to the extent observed in nature‐based VR systems. The number of available studies remains very limited, preventing firm conclusions.

**FIGURE 2:**
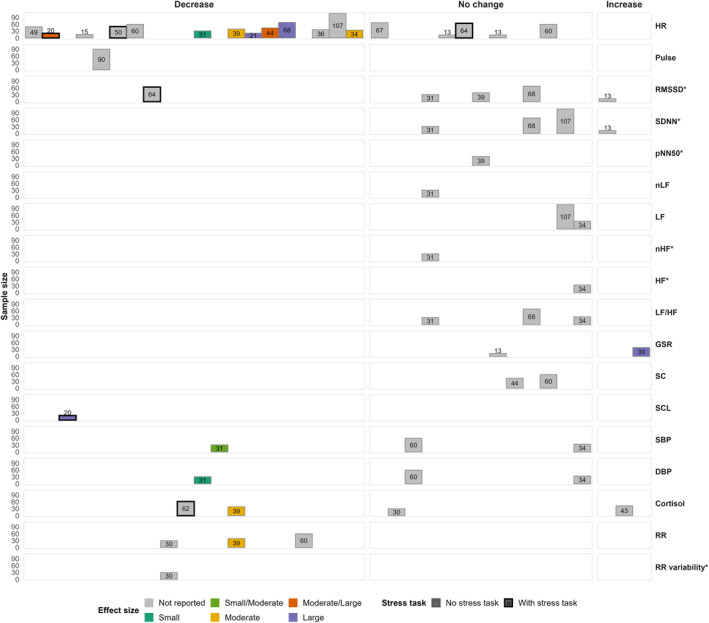
Evidence on the effectiveness of VR stress interventions for pre‐ and post‐intervention assessment of physiological markers. Asterisks (*) indicate markers where an increase is typically associated with relaxation. Each bar represents a study reporting pre‐ and post‐intervention measurements of physiological markers. Studies measuring multiple markers appear across multiple rows vertically. Bar height represents sample size. The border thickness indicates whether a stress task was included in the study design.

Twenty‐six studies incorporated a stress task before VR exposure and 24 studies used a pre‐ and post‐intervention assessment design. In the harvest plot, see Figure 2, reductions in HR were the most consistently reported effect across both subgroups, those with and without stress tasks. For other physiological measures (e.g., RMSSD, SDNN, and cortisol), results did not indicate a consistent pattern related to the presence of stress induction.

Across six studies, two found no effects on either PSS or psychological measures (Cawley and Tejeiro 2024; Jeong et al. 2022), two reported decreases in both PSS and HR (El‐Qirem et al. 2023; Richesin et al. 2021), one observed reduced HR without changes in PSS (Mostajeran 2021), and one reported increase blood pressure despite no PSS effect (Ho et al. 2023), indicating that physiological changes were inconsistently reflected in psychological measures. For PANAS, increases in positive affect (PA) and negative affect (NA) were accompanied by reduced autonomic arousal, reflected in lower HR, BP, RR, and EDA, and increased HRV indices (Anderson et al. 2017; Chan et al. 2023; Gu et al. 2021; Liszio et al. 2018; Richesin et al. 2021; Yuan et al. 2022), or no physiological effects (Ho et al. 2023; Sun et al. 2023). One study found a reduction in PA, NA and HR (Naylor et al. 2019). Other studies reported no change in overall PANAS scores despite physiological improvements (Liszio and Masuch 2019; Villani and Riva 2008). Across studies, most reported decreases in STAI accompanied by reductions in HR or RR and increases in HRV indices (Aganov et al. 2020; Blum et al. 2019; Liszio et al. 2018; Liszio and Masuch 2019; Pardini et al. 2024; Richesin et al. 2021; Rockstroh et al. 2020; Villani and Riva 2008, 2012; Yin et al. 2020), while cortisol and EDA showed no change (Chavez et al. 2020; Pallavicini et al. 2009). While physiological and psychological measures are routinely collected together, they are rarely examined to one another. For example, of the 16 studies that collected both physiological data and PSS, 13 studies did not examine their relationship (PSS: 13/16; PANAS: 13/14; STAI: 15/17). The field has not established clear frameworks and designs for understanding their relationship or how they together provide an indication of the multidimensional stress response. When correlations were examined, they consistently revealed weak or absent relationships between subjective and physiological changes, suggesting these domains may capture distinct aspects of stress responses that do not necessarily align.

While 56 studies indicated that changes in physiological markers are associated with relaxation following VR stress intervention, only a few studies controlled for cofactors such as time‐of‐day, caffeine intake, or motion sickness, which may contribute to the observed variance in outcomes. Some studies suggested that the absence of observed changes could be due to factors such as physical movement or excitement (Ahmaniemi et al. 2017; I. Kim et al. 2023), participants being healthy (Jeong et al. 2022), or the absence of stress induction (Kamińska et al. 2020).

## 4 Discussion

### 4.1 Summary of Main Findings

This review synthesised findings from 69 studies examining physiological markers in VR‐based stress interventions. Many studies reported small but significant shifts in autonomic nervous system (ANS) activity, resulting in decreased sympathetic activity (e.g., reduced heart rate (HR) and skin conductance levels (SCL)) and increased parasympathetic activity (e.g., higher heart rate variability (HRV)). Most studies employed nature‐based VR experiences, and 26 studies (38%) incorporated a stress‐inducing task before the intervention. Despite promising results, particularly consistent reductions in HR observed in single‐session, laboratory‐based assessment using nature‐based VR interventions in general population settings, substantial inconsistencies in study design, such as sample size, measurement protocol, intervention duration, and control of cofactors (e.g., time‐of‐day), contribute to heterogeneity across studies and complicate the interpretation of physiological outcomes.

### 4.2 Interpretation and Methodological Limitations

Consistent with prior research, the findings suggest that both parasympathetic and sympathetic markers respond to virtual natural environments (Annerstedt et al. 2013; H. Jo et al. 2019; Shirtcliff et al. 2024). Most virtual natural environments evoke restorative effects that align with real‐world nature (Shuda et al. 2020). Commonly used physiological markers include HR, root mean square of successive differences (RMSSD), standard deviation of NN intervals (SDNN), galvanic skin response (GSR), SCL, and respiration rate. SCL, a measure of sympathetic nervous system activity, often showed decreasing patterns in virtual nature, indicating reduced physiological arousal (Manaf et al. 2021; Richesin et al. 2021). HRV parameters, which primarily reflect parasympathetic activity, tend to increase, while HR decreases, supporting the calming effects of virtual nature (Riches et al. 2021; Shaffer and Ginsberg 2017).

Despite these trends, methodological heterogeneity, particularly in how physiological markers are measured and reported, limits theoretical integration. Variations in HRV indices, recording duration, and contextual conditions complicate interpretation. Standardisation of protocols is essential for drawing valid conclusions about ANS activity (Quigley et al. 2024). Moreover, many studies prioritise technological innovations over grounding the physiological markers in well‐established stress theories, such as the neurovisceral integration model (Thayer and Lane 2000) or the psychophysiological coherence model (McCraty and Bradley 2009). This has led to a fragmented field in which physiological outcomes are often reported without a clear theoretical rationale for their selection or interpretation. Addressing these methodological and theoretical inconsistencies is crucial for advancing the evidence base of VR stress research and ensuring its effective translation into real‐world health and clinical applications.

The results indicate limited consideration of individual differences in autonomic regulation, further complicating theoretical coherence. Baseline physiological states can significantly influence the potential for observable effects. Factors such as age (Voss et al. 2009), environment (Fatisson et al. 2016), and lifestyle (Karpyak et al. 2014) affect stress responses. Stress responses emerge from interactions between individual appraisals and environmental stressors, influencing subjective, cognitive, behavioural, and physiological outcomes (Carl et al. 2019). Accounting for these differences is crucial when interpreting HR and HRV data (Quigley et al. 2024). Beyond individual variability, the frequent discordance between physiological and subjective measures has important interpretative implications. Physiological changes in autonomic function do not consistently reflect or predict changes in subjective stress, which aligns with previous theoretical work (LeDoux and Pine 2016). This suggests that physiological and subjective measures arise from multiple stress response systems that do not necessarily align in time or magnitude. However, many of the included studies implicitly treat physiological and psychological indices as interchangeable or convergent indicators of stress responses. Adopting a more integrated framework that incorporates individual variability, aligns with stress theory, and combines physiological monitoring with subjective measures within a complementary, multimodal assessment approach would improve the reliability of VR stress research.

Additionally, 26 studies used a stress task preceding the VR stress intervention. A small subgroup comparison between studies with and without stress induction, using a pre‐ and post‐intervention design, revealed that the inclusion of a stress task did not systematically alter outcomes on physiological markers. This suggests that while VR interventions appear to support relaxation and recovery, these effects may partly reflect natural rebound processes rather than stimulus‐specific physiological changes or specific relaxing effects of the VR intervention itself. Such rebound effects, where physiological markers return to baseline or below due to homoeostatic regulation, have been well documented (Campbell and Ehlert 2012). While this design can amplify measurable relaxation effects, it may also overestimate the efficacy of VR interventions. This concern aligns with the findings from a meta‐analysis on VR exposure therapy, which emphasised the importance of considering baseline stress levels (Carl et al. 2019). While this design enhances measurable effects, it may limit generalisability to real‐world settings where stressors are not artificially induced. VR interventions may show limited effects without prior stress induction, especially in participants with low baseline stress.

Several limitations should be considered. First, most studies were conducted in controlled laboratory settings, though interest in real‐world applications is growing. Second, few studies in the professional and mental healthcare setting were identified, despite a specific interest in this area due to the review's focus on advancements in VR interventions. Third, heterogeneity in study design, interventions, and outcome measures makes comparisons difficult and limits the reliability of conclusions. Finally, the review included studies regardless of design quality. While this broader approach offers a comprehensive overview, many studies had small sample sizes and low methodological rigour, warranting cautious interpretation of the outcomes.

### 4.3 Recommendations

To prevent an unwanted proliferation of VR stress interventions, standardized physiological marker selection and measurement protocols should be used in future research. Physiological markers, such as HR and HRV (e.g., RMSSD), are most commonly used, easy to measure continuously, and seem to indicate changes related to stress reduction. However, there was not enough evidence to recommend non‐linear HRV measures, respiratory rate, cortisol, systolic blood pressure (SBP), and diastolic blood pressure (DBP), or non‐commonly used markers, such as IBI. In the professional and mental healthcare settings, larger, longitudinal, and controlled studies should be performed. Integrating individual variability and aligning measurement protocols with established stress theories, such as the neurovisceral integration model and the psychophysiological coherence model, could enhance the reliability of VR stress intervention research. Even in usability‐focused studies, explicit theoretical grounding is valuable. Without it, physiological signals are often interpreted through an implicit lens of naïve realism, assuming, for instance, that HRV directly reflects user comfort or stress (Cacioppo et al. 2016). This overlooks the contextual, interpretive, and sometimes paradoxical nature of psychophysiological responses. Grounding analyses in stress regulation theories (e.g., neurovisceral integration) helps avoid uncritical inference and supports more nuanced interpretations of user experience.

In the design of the studies, a mixed design, combining between‐subjects and within‐subjects approaches, is beneficial to account for individual variability, initial stress levels, and specific physiological patterns that could influence the outcomes. Additionally, measuring both psychological and physiological outcomes, measuring physiological markers at least three times, at baseline, during VR, and after VR, providing the VR intervention for an extended period of a couple of weeks, and accounting for confounding and context variables are essential parts of designing studies. Critically, studies should explicitly examine relationships between physiological and subjective outcomes, as these domains of stress responses often show weak or absent correlations and cannot serve as proxies for one another. This approach is essential not only for capturing the full trajectory of the stress response but also for distinguishing between natural recovery and the specific effects of the VR intervention. Recovery is a critical component of the stress response and must be accounted for, ideally through appropriate control conditions and repeated physiological assessments, to draw valid conclusions about intervention efficacy (Linden et al. 1997). Finally, it is important to consider the potential rebound effect after a stress task preceding the VR intervention. To strengthen causal inference, future studies should incorporate rigorous control conditions that are both time‐ and expectancy‐matched (e.g., non‐VR relaxation tasks or sham VR experiences). Such controls are essential for distinguishing between natural recovery, expectancy effects, and the specific contribution of VR. In addition, longitudinal approaches using ambulatory physiological monitoring, including ecological momentary assessment and intensive data collection, are needed to evaluate the efficacy of VR interventions in real‐world contexts and among populations experiencing chronic stress. This could enhance the ecological validity of findings and help determine how psychophysiological responses to VR interventions perform outside of artificially induced stress scenarios and controlled laboratory settings.

### 4.4 Conclusions and Future Directions

Despite the rapid advancements in VR stress interventions and the increasing use of physiological markers, research integrating these two fields and assigning meaning to changes in the markers still lacks standardisation. The findings of this review suggest emerging evidence supporting the use of physiological markers in assessing the effectiveness of VR stress interventions. However, methodological inconsistencies and the absence of standardized protocols make it difficult to draw definitive conclusions about their general effectiveness. To advance the field, future research should focus on aligning physiological measurements with established stress models, enhancing both theoretical and empirical rigour in VR‐based stress research. Personalisation is another crucial factor, as individual variability in physiological responses suggests that a one‐size‐fits‐all approach may not be optimal. Smaller, individually tailored studies can help determine the ideal duration and intensity of VR stress interventions in both controlled laboratory settings and real‐world environments (Maric and van der Werff 2020). Additionally, integrating adaptive algorithms that adjust VR stress interventions based on real‐time physiological feedback may further enhance their efficacy. To facilitate the implementation of VR stress interventions into daily life, future studies should employ longitudinal and ambulatory assessments to evaluate the long‐term benefits and potential limitations compared to conventional methods. Investigating the optimal duration, frequency, and context of VR sessions for diverse populations and stress levels will be essential for broader implementation. Ultimately, addressing these gaps will contribute to a more robust and standardized framework for VR stress interventions, ensuring their effectiveness and applicability in both clinical and everyday settings.

## Funding

ZonMW, Grant Nos. 50‐55515‐98‐011 & 80‐85200‐98‐21015. Matthijs Noordzij receives support from the Stress in Action research programme (www.stress‐in‐action.nl), funded by the Dutch Research Council (NWO Gravitation Grant No. 024.005.010) and the Dutch Ministry of Education, Culture and Science.

## Conflicts of Interest

The authors declare no conflicts of interest.

## Data Availability

Data sharing not applicable to this article as no datasets were generated or analysed during the current study.

## Appendix A

### Search Strategy PubMed:

(“Virtual Reality”[Mesh] OR “virtual realit*”[tiab] OR VR[tiab] OR “Virtual Reality Exposure Therapy”[Mesh]) AND (“Stress, Physiological”[Mesh] OR “Stress, Psychological”[Mesh] OR stress*[tiab] OR “Relaxation Therapy”[Mesh] OR “Relaxation”[Mesh] OR relaxation*[tiab] OR “Respiratory Rate”[Mesh] OR “respiratory rate*”[tiab] OR “Blood Pressure”[Mesh] OR “blood pressure”[tiab] OR “vagal tone*”[tiab] OR “Hydrocortisone”[Mesh] OR hydrocortisone[tiab] OR cortisol[tiab] OR.

“Dehydroepiandrosterone Sulphate”[Mesh] OR dehydroepiandrosterone sulphate[tiab] OR “Electrocardiography”[Mesh] OR electrocardiography[tiab] OR electrodermal[tiab] OR EDA [tiab] OR “Galvanic Skin Response”[Mesh] OR “galvanic skin response”[tiab] OR GSR[tiab] OR “Heart Rate”[Mesh] OR “heart rate*”[tiab] OR HRV[tiab] OR “Hypothalamo‐Hypophyseal System”[Mesh] OR “hypothalamo‐hypophyseal system”[tiab] OR HPA[tiab]) NOT (“Oxidative Stress”[Mesh]).
